# IVF cycle safety when a positive passive air sampling occurs under laminar flow hood in absence of a detectable contamination in the embryo culture

**DOI:** 10.3389/fcell.2024.1474242

**Published:** 2025-01-22

**Authors:** Claudia Omes, Roberto Bassani, Patrizia Cambieri, Fausto Baldanti, Rossella Elena Nappi

**Affiliations:** ^1^ Center for Reproductive Medicine–Obstetrics and Gynecology Unit 2, Woman and Child Health Department, Fondazione IRCCS Policlinico San Matteo, Pavia, Italy; ^2^ Microbiology and Virology Unit, IRCCS Fondazione Policlinico San Matteo, Pavia, Italy; ^3^ Department of Clinical, Surgical, Diagnostic and Pediatric Sciences, University of Pavia, Pavia, Italy

**Keywords:** air quality, embryo culture, clinical outcome, IVF procedures, microbiological contamination

## Abstract

Microbiological contamination in the embryo culture media might affect embryo early development and clinical outcomes during IVF procedures. Infections in the genital tract represent the most common causes of culture contamination, but also environmental air quality might have a detrimental effect on reproductive outcomes of infertile couples undergoing IVF procedures. Monitoring microbiological contamination in an embryology laboratory is mandatory and daily tests are performed under laminar vertical flow hood. In this study, we investigated the IVF outcome of procedures carried out during 5 years of laboratory activity when a positive passive air sampling occurs under laminar flow hood in the absence of clear contamination in the embryo culture. We performed 570 air samplings, and we isolated at least 1 CFU of microorganisms in the TSA settle plate in 13 cases (2.28%). No infections were suspected in the culture media given the absence of detectable microorganisms under the microscope or a turbidity/color change of culture media visible to the naked eye (0% contamination rate). There were no statistically significant differences in biochemical pregnancy, live birth rate, and abortion between the “contaminated” Group P and the “negative” Group N. Surprisingly, we found a better outcome in terms of clinical pregnancy rate in Group P as compared to Group N, a finding likely due to the accidental lower age of Group P (*p* = 0.0133). Data showed that, in the absence of a detectable contamination in the embryo culture media, IVF cycles are safe when an air positive sample occurs in Grade A environment.

## 1 Introduction

The development and growth of *in vitro* fertilization (IVF) techniques in recent decades has led to increasing attention towards the impact that environmental microbiological contamination could have on embryonic development in culture conditions (Mortimer et al., 2018). This interest is reinforced by the inclusion of this topic even in the guidelines for the quality and safety of tissues and cells for human application (EDQM, 2022). Over time, increasing attention has been paid to each minute detail that characterizes an IVF cycle with the aim of improving reproductive outcomes of patients who undergo the procedures, with a special attention on the air quality. Embryologists must be correctly trained to reduce any form of cross-contamination. They should learn the management of a controlled contaminated environment also avoiding risky behaviors. For example, personnel who smoke could introduce toxin compounds into embryo laboratory from their skin or clothes (Hang et al., 2013). Products, furniture and instruments introduced in laboratories should be manufactured with low release VOCs (volatile organic compound) materials (Mortimer et al., 2018) and they must be disinfected and cleaned with low embryo toxicity disinfectants before entering the embryology lab and after routine use (Catt et al., 2013). The environmental contamination should occur under control with HVAC (Heating, Ventilation and Air Conditioning) system to maintain GMP Grade D (ISO 8) as indicated by International consensus (Mortimer et al., 2018; EudraLex, 2008; ISO Technical Committee 209, 2015) and local directives for air background. During the most critical manipulation steps (oocyte pick-up retrieval, decumulation, vitrification, etc.), every activity should be done under the vertical laminar flow hood to maintain a sterile environment; embryo culture under oil is strongly recommended to assure safety of procedures (Scarica et al., 2022). Most studies showed that the principal source of microbiological contamination in embryo culture is represented by human samples (Zheng et al., 2023). The most common contaminant is the semen in 63%–100% of cases (Cottell et al., 2000) and the second one is the follicular fluid, with 9%–27% of positive bacterial culture rate (Štšepetova et al., 2020). For these reasons, the collected samples should be strictly screened by applying the best protocol able to minimize possible cross-contamination. Overall, these containment measures guarantee a risk of contamination detectable in IVF culture dishes that is under 1%, with a variability from 0.24% to 0.69% among studies (Ben-Chetrit et al., 1996; Cottell et al., 1996; Kastrop et al., 2007; Zheng et al., 2023). The monitoring of microbiological contamination includes specific protocols to test air quality also under the vertical laminar flow hood during workflow. In addition to the periodic checks carried out under flow hood as required by GMP Grade A (ISO 5) (EudraLex, 2008; ISO Technical Committee 209, 2015), it would be advisable to carry out routine daily air control with plates of TSA (trypticase soy agar) for passive air sampling in order to verify the absence of disturbances in the air quality. Plates should be incubated for 3 days at 20°C–25°C followed by incubation at 30°C–35°C for additional 2 days. This protocol has been shown to be adequate to detect most bacteria and fungi (EDQM, 2022; WHO, 2012). Most of the studies reported in the literature showed cases where the contamination is evident in embryo culture dishes for cloudiness and/or color change of the culture media. In these cases, the effects on embryo development are evident showing an arrest of cleavage and an increasing risk of cycle cancellation, resulting in increased costs, and reducing patient confidence (Borges et al., 2020). It is very difficult to define the effect of air contamination when it is not detectable in the culture media because the embryo develops as usually expected. Although it is important to constantly monitor air quality, the time necessary to detect a contamination issue is long because microbiological plate incubation lasts up to 5 days. Therefore, it is not possible to intervene during embryo culture development because microbiological results will become available after cell use or implantation (EDQM, 2022). Embryos correctly reaching cleavage or blastocyst stage are usually transferred to the uterus, whereas supernumerary embryos are cryopreserved and transferred subsequently if pregnancy is not achieved at first attempt. The lack of knowledge about microbiological results of passive air sampling before the clinical use of cells generates a question marker in the management of the IVF procedure both from practical and ethical stand points. In the present study, we investigated the safety of IVF procedures when a positive passive air sampling occurs accidently under laminar flow hood during workflow of the laboratory of embryology, in the absence of detectable contamination in the embryo culture.

## 2 Materials and methods

### 2.1 Study setting and design

This retrospective study included IVF cycles with at least one transfer (n = 1,223: fresh n = 726; and frozen n = 497) carried out between January 2018 and December 2022 at the Center for Reproductive Medicine - Obstetrics and Gynecology Unit 2 (Woman and Child Health Department, Fondazione IRCCS Policlinico San Matteo, Pavia, Italy). This study was conducted according to the guidelines of the Ethics Committee of IRCCS San Matteo Foundation. Written informed consent was obtained from each subject involved in the study for the use of personal data. The results of air sampling done during the study period were reported and clinical outcomes (biochemical pregnancy, clinical pregnancy, live birth rate and abortion) were considered.

According to GMP and ISO rules (EudraLex, 2008; ISO Technical Committee 209, 2015), every 6 months, air quality of biohazard vertical flow hood is controlled for monitoring particle concentration and microbiological contamination by a specialized company. To guarantee a continued monitoring of air quality, during daily routinely procedures, other tests are performed by embryologists. Monitoring is carried out during the activity using a sedimentation plate of TSA (90 mm diameter) for the entire duration of the processing, if this is less than 4 h. If the processing requires longer times, the monitoring should last at least 4 h (parameter: CFU/4 h). The embryologist staff took care of sending the samples to the microbiological laboratory unit of the hospital.

### 2.2 Plate incubation and microbiological sample results

Plates should be incubated as quickly as possible to ensure that the microorganisms remain viable. The plates, after having been closed with a flexible thermoplastic to avoid external contamination, must be transported as quickly as possible to the microbiology laboratory. The plate culture media must be accompanied by growth promotion test and suitability of method test certificates. The incubation included 3 days at 20°C–25°C followed by a further incubation at 30°C–35°C for another 2 days to detect most bacteria and fungi. Results were reported as number of CFU/4 h that should be <1 for GMP Grade A. When a contamination occurred, microorganisms of colony grown on the TSA were identified to the genus and species level with mass spectrometry MALDI-TOF. All bacterial strains were preserved for further investigation.

## 3 Results

**FIGURE 1:**
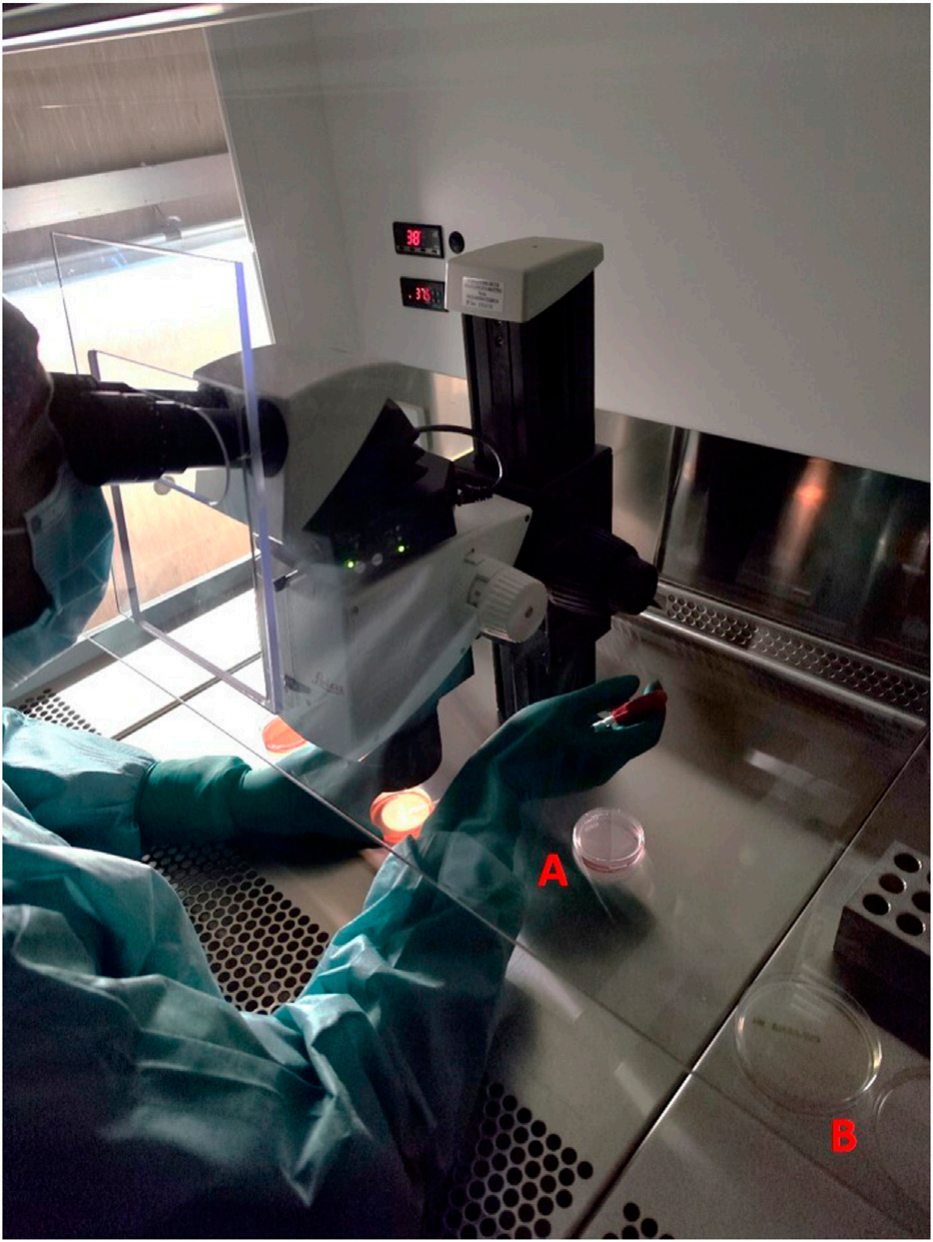
Example of a typical setting in the IVF lab under vertical flow hood during oocyte pick-up. The embryologist is isolating oocytes from follicular fluid under microscope and transfer them into culture media **(A)** while TSA plate for passive air sampling for sedimentation is in the workflow **(B)**.

**FIGURE 2:**
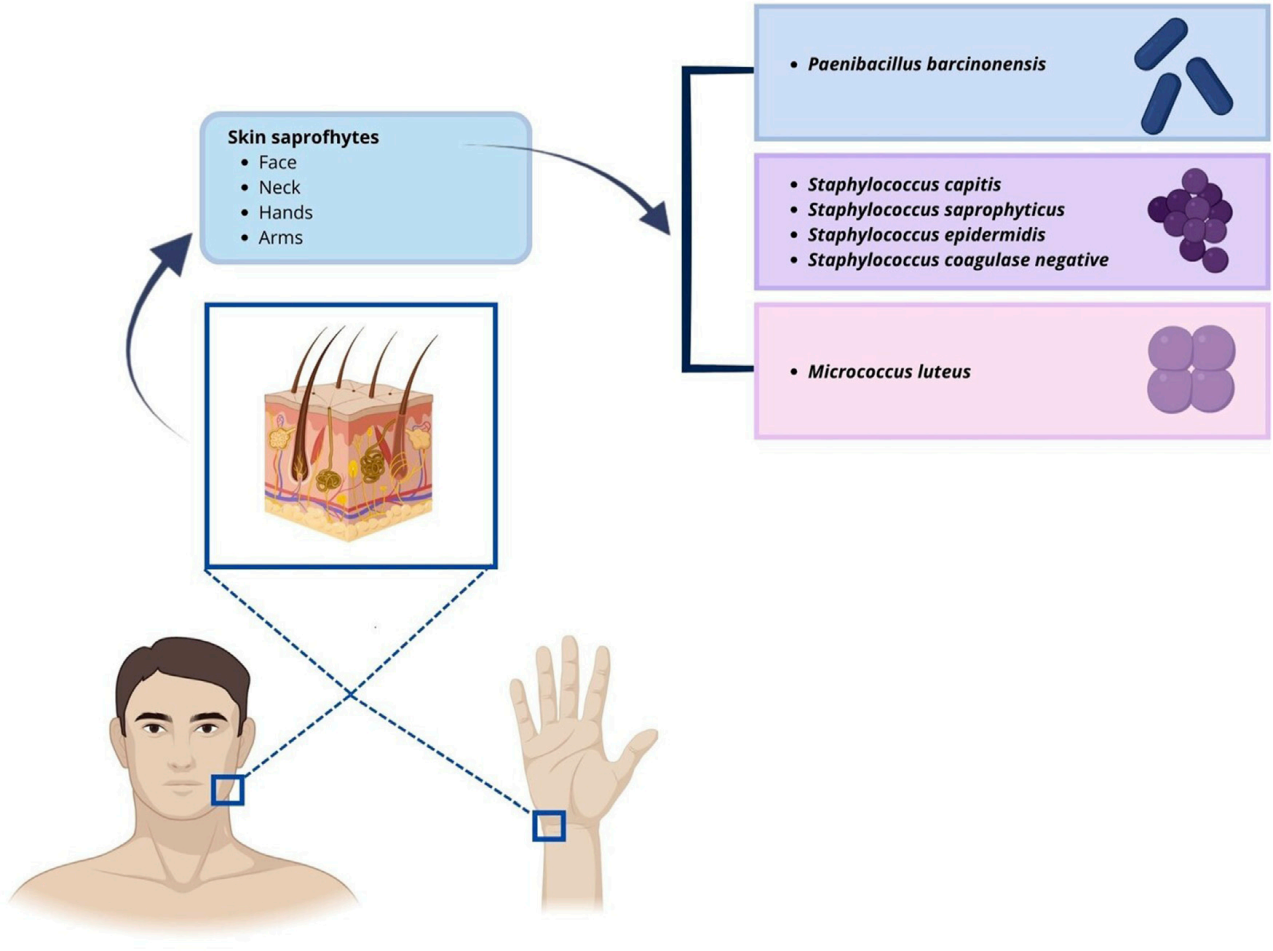
Illustration of microorganisms identified in positive passive air sampling and their potential sites of origin.

Between 2018 and 2022, 1223 IVF cycles with at least one embryo transfer were carried out. Results of air quality tests done routinely in the embryology lab under flow hood during workflow were reported. In the culture media of oocytes and embryos, there was no evidence of obvious contaminations because a detectable presence of microorganisms under the microscope or a turbidity/color change of culture media, that was visible to the naked eye (0% contamination rate), has been never observed. Air samplings performed during the workflow have been obtained by the exposition of TSA plate under flow hood during routine IVF lab procedures to collect microorganisms circulating in the air, which fall due to the sedimentation as showed Figure 1. We analyzed 570 air sampling performed under flow hood during workflow of IVF procedures in 5 years of our activity. Although a contamination of embryo culture was not detectable in TSA plates used for passive air sampling for sedimentation, we found at least 1 CFU/4 h in 13 cases (2.28% positivity). The report of positive air sampling and microorganism isolated is shown in Table 1. In three cases, it was impossible to complete microorganism identification. Misidentifications often occur with environmental microorganisms outside the specific protein spectrum included in the clinical library. With MALDI-TOF MS technology, the characterization of microorganisms derives from the “mass spectra” comparison with fingerprints database. The spectrum obtained is compared with the reference ones previously collected and deposited in commercially available libraries. In the 76.92% of positive air samplings, we were able to identify contaminant microorganisms, which were always represented by saprophytes of the skin (Figure 2). Due to the origin of the bacteria detected, movements of the embryologists under the hood represent the most probable cause of contamination. Indeed, possible environmental contaminants should correctly be filtered by the vertical laminar flow hood, ensuring complete protection. Accidental contamination highlighted on the air test control plates could also occur simply when the plates were opened under the hood or when they were closed before being sent to the microbiology laboratory. In the absence of a detectable contamination of the embryo culture media or without a control test performed in the spent media at the end of embryo culture it was not possible to confirm any contamination in the IVF procedure. Reproductive outcomes of the cycles were considered in order to investigate possible negative effects on the embryo culture due to the detection of positive air samplings under flow hood. The 50 cycles carried out during contamination (Group P) were compared to the 1,173 cycles completed when all air samplings performed when gametes or embryos were manipulated under flow hood resulted negative (Group N). We considered as “contaminated” (Group P), every cycle in which even a single phase was judged at risk (oocyte pick-up, decumulation, transfer, vitrification, thawing, fertilization check, etc.) for the detection of at least one colony in the TSA plates. Biochemical pregnancy rate, clinical pregnancy rate, spontaneous abortion rate, live birth rate and birth defect rate at birth were considered (Table 2). No statistically significant differences emerged in the analysis of reproductive clinical outcomes between the two groups except for a better statistically significant clinical pregnancy rate in the Group P (36.00% vs. 22.17%, *p*-value = 0.0345). However, this unexpected result was probably due to the lower significant age of patients in Group P (34.48 ± 5.00 years) as compared to Group N (36.32 ± 4.54 years) (*t*-test: *p*-value = 0.0133). Data collected confirmed the safety of IVF cycle when a positive passive air sampling accidently occurs under laminar flow hood in absence of a detectable contamination revealed in the embryo culture media.

**TABLE 1:** Prevalence of contaminants detected in the positive passive air sampling under flow hood during workflow.

Microorganism identification	No. samples	No. CFU/sample	Frequency of isolation
*Paenibacillus barcinonensis*	1	2	7.69% (1/13)
*Staphylococcus capitis*	1	2	7.69% (1/13)
*Staphylococcus saprophyticus*	1	22	7.69% (1/13)
*Staphylococcus epidermidis*	1	1	7.69% (1/13)
*Staphylococcus* coagulase negative	2	1	15.38% (2/13)
		2	
*Micrococcus luteus*	4	1	30.77% (4/13)
		1	
		4	
		1	
Not identificable	3	1	23.08% (3/13)
		6	
		1	

**TABLE 2:** Analysis of the clinical outcomes of IVF cycles carried out when a passive air sampling results incidentally positive.

Indicator	Positive air sampling detection (Group P)	Negative air sampling detection (Group N)	X Chi^2^ with Yates correction	P-value
Cycles	50	1173	—	—
Biochemical pregnancy rate (%)	8.00% (4/50)	8.44% (99/1173)	0.0226	0.8805
Clinical pregnancy rate (%)	36.00% (18/50)	22.17% (260/1173)	4.4678	0.0345*
Spontaneous abortion rate (%)	16.67% (3/18)	19.62% (51/260)	0.0000	0.9982
Live birth rate (%)	28.00% (14/50)	17.31% (203/1173)	3.0606	0.0802
Birth defect rate (%)	0% (0/16)	0.004% (1/227)	—	—

*< 0.05 Chi-square test with Yates correction.

## 4 Discussion

During IVF procedures both guarantee and security of the protocols applied are fundamental to assure the best chances of achieving pregnancy in infertile couples. The procurement and processing of reproductive cells and human embryos should be subjected to comprehensive risk assessment to allow identification of those steps, which need the highest rate of controls and required validation (EDQM, 2022). The microbiological contamination of the embryo culture system represents one of the risks that should be avoided. The main cause of this accidental contamination is due to the characteristics of biological samples under manipulation. It is known that genital tract pathogens could be associated with IVF failure, leading to early pregnancy loss or preterm birth (Ricci et al., 2018). Although most contamination could occur in follicular fluid or semen (Zheng et al., 2023), it is not rare to observe accidental contamination, even under adequate surveillance in IVF laboratories (Kastrop et al., 2007; Borges et al., 2020). This condition arises, namely, because of air quality or personnel manipulation (Mortimer et al., 2018). That being so, it is mandatory to perform microbiological monitoring with different sampling methods as volumetric air sampling, settle plates, contact plates and glove prints (EDQM, 2022). The frequency of sampling should take into account the volume of activities. For example, aseptic workflow performed in a Grade A environment must be monitored routinely, during every process (EDQM, 2022). In this study, data on the results of air daily monitoring under flow hood were collected retrospectively to evaluate the possible impact on IVF outcomes when microbiological analysis revealed a contamination that did not correspond to a detectable microbiological growth in the embryo culture media. During 5 years of observation, we did not find any contamination directly in the embryo culture media. However, we did find the settle plate used to monitor the aseptic workflow under flow hood contaminated with microorganisms on 13 occasions. In all cases, isolated microorganisms were normally skin saprophytes, probably derived from accidental personnel contamination. We considered IVF cycles performed during the days in which contamination was detected. Any statistically significant difference was found in terms of reproductive outcomes in IVF cycles carried out when air sampling under flow hood resulted positive or not. These data suggest that IVF procedures have shown the expected outcomes, ensuring their safety, also when a positive passive air sampling occurs under a laminar flow hood in the absence of detectable contamination in the embryo culture. IVF culture media contain penicillin and streptomycin that limit a possible growth of microorganisms if they are not resistant to these antibiotics. The detection of a positive air sampling in the settle plate in a Grade A environment does not necessarily correspond to real contamination in the culture media because it could be due simply to the carelessness of the operator during the opening and closing of the TSA plates. If a colony forming unit accidently falls into the culture medium containing embryos, in most cases this does not seem to cause growth of the microorganism (Kastrop et al., 2007), which is blocked by the antibiotic, and therefore no effects are induced on the embryo development and IVF outcome. Our results demonstrate that the accuracy of personnel during IVF workflow is one of the fundamental aspects to bear in mind in evaluating successful outcomes of laboratory procedures. On the other hand, in a Grade A environment daily controls of air quality might be redundant. A more effective strategy could be performing daily control in a first phase to validate laboratory workflow and then reduce the frequency of monitoring, turning back to the previous frequency in case some positivity will manifest. Particular attention should certainly be paid in case a new member of the staff is introduced. Indeed, the training of new operators in an IVF laboratory is long and complex, and there is still some debate on the best method to certify competence acquired as well as number of staff necessary to assure optimal safety and efficiency of operations (ESHRE et al., 2016; Kovacic et al., 2020; Veiga et al., 2022; Sciorio et al., 2022). Among the skills needed for trained embryologists, special attention should be paid to their ability to move and manipulate cells in the delicate IVF environment in which air quality is so fundamental (Mortimer et al., 2018). In addition, when a new component of IVF staff laboratory has to be introduced it is recommended to increase the air sampling under flow hood to daily frequency for a period that could document the ability acquired by the new operator to correctly move in the IVF laboratory environment minimizing the risk of microbiological contamination. To guarantee the absence of contamination during embryo culture, a future study could be conducted to test the sterility of spent culture media after all embryos have been transferred, cryopreserved, or disposed of.

## Data Availability

The original contributions presented in the study are included in the article/supplementary material, further inquiries can be directed to the corresponding author.
